# The Dominance of Blended Emotions: A Qualitative Study of Elementary Teachers’ Emotions Related to Mathematics Teaching

**DOI:** 10.3389/fpsyg.2020.01865

**Published:** 2020-08-06

**Authors:** Dionne Indera Cross Francis, Ji Hong, Jinqing Liu, Ayfer Eker, Kemol Lloyd, Pavneet Kaur Bharaj, MiHyun Jeon

**Affiliations:** ^1^Culture, Curriculum and Teacher Education, University of North Carolina at Chapel Hill, Chapel Hill, NC, United States; ^2^Department of Educational Psychology, The University of Oklahoma, Norman, OK, United States; ^3^Department of Curriculum and Instruction, Indiana University, Bloomington, IN, United States; ^4^Faculty of Education, Giresun University, Giresun, Turkey

**Keywords:** mixed emotions, mathematics teacher emotions, teacher emotions, mathematics teacher quality, teaching quality, blended emotions, emotion blends

## Abstract

Examining the nature of teachers’ emotions and how they are managed and regulated in the act of teaching is crucial to assess the quality of teachers’ instruction. Despite the essential role emotions play in teachers’ lives and instruction, research on teachers’ emotions has not paid much attention on teachers’ emotions in the context of daily teaching. This paper explored elementary teachers’ emotions while preparing for teaching and during teaching mathematics, reasons that underlie these emotions, and the relationship between their emotions and the quality of their mathematics instruction. Participants were seven elementary teachers working in the U.S. who participated in Holistic Individualized Coaching (HIC) professional development that consisted of five cycles of coaching over an year. For each coaching cycle, pre-coaching conversation and post-coaching conversation data were collected regarding emotions teachers felt in anticipation of teaching and during teaching retrospectively. In order to compare teachers’ emotions with instructional quality, coaching sessions were video recorded and analyzed to determine the quality of instruction. Findings of this study showed that teachers reported six categories of emotions (positive, negative, neutral, blended-positive, blended-negative, and mixed), described emotions often in non-typical ways (e.g., “not nervous”, “anxious but in a positive way”), and experienced mixed emotions (co-occcurence of positive and negative emotions) as the most dominant emotion. Teachers also had more positive emotions anticipating teaching than actually teaching the lesson. The reason teachers felt mixed emotions reflected the complex and context-specific nature of teaching, a phenonemenon not currently described in the teacher emotion literature. There were no clear relationships between emotional experiences and instructional quality. This study allowed participants to freely describe their authentic, complex, overlapping, and ambiguous emotions in the context of active teaching, which contributes opening up the possibilities of diversifying teacher emotion research and shows the significance and usefulness of understanding teachers’ emotions related to active instruction.

## Introduction

Emotions are an omnipresent aspect of teachers’ daily experiences (Nias, 1996; Hargreaves, 2000; Sutton and Wheatley, 2003). They are not peripheral; in fact, they are integrally connected to cognition and action and as such guide the processes that inform how we make choices and act (Oatley, 1991; Hargreaves, 2000). They also guide our judgment and frame what and how we reflect. In this regard, teaching and learning can be seen as emotional practices (cf. Denzin, 1984), as they engender feelings within ourselves and within those with whom we interact, namely, students. Recognizing the integral role emotions play in teachers’ lives, research on teachers’ emotions has steadily increased over the last few decades. However, relative to the other foci on teacher research, for example, teacher learning, research on teacher emotions can still be regarded as emerging. What tends to be foregrounded in teacher research is the instructional work of teachers and its relation to student outcomes, primarily academic achievement and well-being (including identity, efficacy, and social–emotional competencies) (Rosiek, 2003). Teachers’ own psychological lives, or the ways that teachers’ practices and emotions are intertwined, have been less of a focus (Frenzel, 2014), to the extent that “being tactful, caring or passionate as a teacher is treated as largely a matter of personal disposition, moral commitment or private virtue, rather than of how particular ways of organizing teaching shape teachers’ emotional experiences” (Hargreaves, 2001, p. 813). One way in which this disconnects between teachers’ affective and psychological lives and their professional lives is in how teacher knowledge is defined (Zembylas, 2007). In particular, since Shulman’s (1987) work on pedagogical content knowledge, numerous researchers (e.g., Hill et al., 2008) have built on and expanded this work, yet the literature is still sparse related to understanding the emotional dimensions of teachers’ knowledge and work (Zembylas, 2007).

Although research around teachers’ emotions in this and other related areas are growing (e.g., well-being; Keller et al., 2014), an area that has gotten little traction is teachers’ emotions while preparing for teaching (Frenzel, 2014), reflective accounts on teachers’ emotions during teaching, and the relationship between teachers’ emotions and instructional quality. In particular, questions about the kinds of emotions teachers experience related to instruction, and whether these emotions are discrete or dimensional (cf. Barrett, 1998), single or blended (multiple co-occurring emotions, cf. Scherer, 1998), have mainly gone unanswered. As such, in this study, we address this gap. Specifically, we focus on describing elementary teachers’ emotions in relation to their mathematics teaching, examine the complexity of the emotional occurrences–whether independent or blended, the reasons that underlie these emotions, and the relationship between these emotions and the quality of their mathematics teaching.

## Teachers’ Emotions

In this section, we describe how emotions are categorized and the theoretical model for how emotions are elicited and provide a summary of the literature about the antecedents of emotions.

### Theoretical Model of Emotions

Acknowledging both the social and cognitive dimensions of emotions, we draw on Schutz et al.’s (2006) definition of emotions as “socially constructed, personally enacted ways of being that emerge from conscious and/or unconscious judgments regarding perceived successes at attaining goals or maintaining standards or beliefs during transactions as part of social-historical contexts” (p. 344). From this perspective, emotions are considered to be socially constructed and relational, meaning they involve relationships between a subject and a particular object, such as one is frustrated with someone or excited about something (Denzin, 1984; Lazarus, 1991). In this regard, interactions between the person and the environment are necessary for emotional experiences to occur. With teachers, these person–environment interactions tend to occur in their classrooms, as they make efforts to achieve their classroom goals and develop relationships with their students, which tend to be multifaceted.

Goal setting is a central aspect of teaching as teachers’ actions, strategies, and decision-making are guided by their goals (Schutz et al., 2001, 2020). Goals refer to “the object or aim of action” (Locke and Latham, 2013, p. 4). They can be seen as performance outcomes or targets that individuals use to evaluate progress (Pintrich, 2000). Motivation theories suggest that individuals initiate and persist with actions and behaviors to the extent that they believe the enactment of these actions will result in these desired goals or outcomes (Schutz, 1991; Ford, 1992; Deci and Ryan, 2000). So, classroom goals shape teachers’ actions, the decision-making process, the level of effort and energy invested, and quality of the performance. For teachers, goal-setting can be particularly complex because there tend to be multiple types of goals related to each lesson, with each broad goal encompassing subgoals. Camp (2017) found that college teachers have three categories of goals: (i) content goals–related to the specific conceptual ideas they wanted the students to master, or to developing strategies needed to master the content, (ii) course management goals–related to the design and overall administration of the course, and (iii) teaching goals–related to the teaching practices that need to be enacted to support student learning. With respect to K-12 teachers, an additional goal tends to be classroom management, which relates to ways of organizing the classroom and activities to effectively manage students’ behaviors (Sun, 2015; Kwok, 2019). Student misbehavior is often a stressor, so attending to it effectively is often a primary classroom goal for many teachers. Within the classroom context, these multiple goals are concurrently activated through the different stages of teaching–during lesson preparation and development, lesson enactment, and lesson reflection.

These classroom goals tend to function as reference points where teachers make judgments, consciously or unconsciously, about where they are relative to the goal. In so doing, teachers are continuously assessing and interpreting whether or not and how classroom interactions are aiding or hindering attaining these goals (Schutz et al., 2020). These judgments about whether classroom transactions will facilitate goal attainment are called appraisals (Lazarus, 1991, 1999). As Lazarus (1991) noted, emotions are elicited based on the individual’s (in this case the teacher’s) appraisals about perceived success at attaining their goals. For example, if a teacher appraises that the classroom transactions are supportive of her attaining the classroom goal, then positive emotions may be elicited. In contrast, if what is unfolding in the classroom is hindering goal attainment, then negative emotions may be elicited.

As these multiple goals are activated through the teaching process and teachers act in ways to achieve these goals, the appraisal process and emotional experiences are also likely to be multidimensional. We project that these transactions may unfold in three ways. First, teachers may appraise that they are progressing toward achieving all the goals set, thus multiple positive emotions may be elicited. Second, teachers may appraise that progress toward all the goals have been thwarted, thereby eliciting multiple negative emotions. Third, teachers may appraise that they are on track to achieve some goals and not others. In this situation, a combination of positive and negative emotions may be experienced. For example, let’s consider a first-grade teacher has a content goal for students to be able to solve addition problems using a strategy of “*doubles plus one*” supported by the use of an instructional resource called ten-frames. The teacher decides to use the think–pair–share strategy to both support discourse and manage student behavior by keeping students on task. However, as the lesson unfolds, she realizes that students are grasping the concept really quickly, some are finished with the tasks and are beginning to get disruptive. Assessing the situation, she appraises that she has made adequate progress toward her content goal and is excited about her students’ thinking and that she has achieved her content goal. However, the class has quickly unfolded into chaos as most of the students have correctly solved all the problems. Having not anticipated that they would complete the tasks this quickly, she has no additional work ready to reengage the students. She appraises that her classroom and course management goals have been thwarted–she feels angry about the students’ behaviors and anxious about what to do next. Given that she is concurrently appraising multiple goals, multiple emotions (e.g., excitement, anger, and anxiety) are elicited. In this case, there was the co-occurrence of multiple emotions–both positive and negative; however, depending on the appraisal processes, a mixture of positive emotions or a mixture of negative emotions could co-occur.

### Mixed Emotions and Blended Emotions

The co-occurrence of multiple emotions has been hotly debated in the field of psychology for years (Berrios et al., 2015). Emotions are complex experiences, such that research results have shown that not only can multiple emotions co-occur, but also it is possible that individuals can experience two opposite valenced emotions at the same time (e.g., feeling happy and sad)–what is referred to as *mixed emotion* (Larsen et al., 2001). Mixed emotions fall within a broader category of emotions called *emotion blends* (also referred to as *blended emotions*). Emotion blends, or blended emotions, is a more general categorization of emotional experiences combining more than one emotion, but not necessarily emotions of opposite valence (Scherer, 1998). So, the co-occurrence of excitement and pride would be an example of an emotion blend. Davis et al. (2004) observed during stressful times that people tended to experience more blended emotions than single emotions. Emotion blends, as defined, are less controversial in the emotion literature, as the co-occurrence of emotions with similar valence (e.g., happiness and excitement) tends to be less contentious (Berrios et al., 2015). However, questions still linger about whether two opposite valenced emotions (e.g., fear and hope) can co-occur (Lindquist and Barrett, 2008). Critiques of blended and mixed emotions tend to be rooted in the underlying theory of emotions to which the researcher ascribes, such that there is alignment with the Evaluative Space Model (Cacioppo et al., 2004) and Appraisal Theory (Lazarus, 1991), but not with the typical articulation of the Discrete or Basic emotions (Izard, 1972) and Circumplex Models (Russell, 1980).

Blended emotions appear to be compatible with appraisal theory described above. An appraisal is the individual’s evaluation of how a person–environment transaction is progressing–the process is a “link between the organism and the situation that produces the emotion” (Ellsworth and Scherer, 2003, p. 574). In accounting for appraisal processes during emotionally complex experiences, there are evidences to suggest that appraisals can be combined flexibly (Berrios, 2019). In particular, Smith and Ellsworth (1987) examined appraisals and emotions during test taking and observed that different appraisals combined, or combinations of patterns of appraisals, produced emotion blends. Frijda et al. (1989) also observed multiple cognitive appraisals when *being-moved* (a complex emotional experience typically occurring during sentimental life events), including pleasantness, certainty, suddenness, importance, and agency. Emotional blends are accounted for by the versatility of the affective system–its flexibility in allowing people to “combine, aggregate, and fluctuate between different emotions” (Berrios, 2019, p. 4).

### Positive and Negative Emotions

Some of the key discrete emotions that are most predominant in the literature are enjoyment, pride, anger, anxiety, shame, and guilt. Enjoyment and pride are perhaps the two most dominant positive emotions teachers describe related to the classroom (Sutton and Wheatley, 2003; Frenzel, 2014). Enjoyment is the subjective feeling of pleasure connected to an activity or experience thought to be generated from feelings of being in control of a situation that is highly valued (Pekrun, 2006) or from the anticipation of, participation in, or reflection on a desired event or activity (Frenzel, 2014). Given that for most current studies researchers rely on self-report measures, it is possible that teachers may exaggerate their feelings of enjoyment, as it is socially desirable to enjoy teaching and love your students (Winograd, 2003). In this regard, teachers may experience emotional labor due to the inexplicit display rules that guide how teachers think they are supposed to feel and portray emotion. Pride is also commonly experienced by teachers–perhaps second to enjoyment (Frenzel, 2014). It is considered to be a positive emotion aligned with enjoyment that reflects accomplishments that are personal or by others which whom one feels an association.

With respect to negative emotions, anger is one of the most highly reported emotions (Sutton and Wheatley, 2003). In contrast to enjoyment, anger is not socially desirable and as such may influence teachers’ self-reports of anger. So, although it is one of the predominant emotions reported by teachers, because it is negatively viewed, it may be reported at a lower frequency than actually experienced (Sutton and Wheatley, 2003). Research related to teachers’ anxiety is abundant, especially related to test anxiety in students (Sarason et al., 1990; McDonald, 2001) and specifically related to mathematics–referred to as math anxiety (Boaler, 2014). Math anxiety in particular is considered an unpleasant feeling that arises during instances of thinking about or doing mathematics (Hembree, 1990; Ashcraft, 2002; Ganley et al., 2019) and has been associated with reduced performance. Within the increased focus on teachers’ emotions, researchers (Ganley et al., 2019; Olson and Stoehr, 2019) have recognized math anxiety as significant factor in the lives of teachers, especially elementary teachers, and its association with teaching behavior. Elementary teachers tend to have higher levels of math anxiety possibly because they do not obtain higher degrees in math (Hembree, 1990). However, the reason they do not pursue math at advanced levels may be due to the tendency of avoiding situations where they have to do math. Some have hypothesized that there is a gender-related component, as a majority of elementary teachers are women, and women tend to have higher levels of math anxiety (United States Department of Education, 2017).

Some have conceptualized a distinct form of math anxiety–anxiety about teaching math (Peker, 2009; Brown et al., 2011). Peker (2009) defined this type of anxiety as experienced by pre-service and in-service teachers as “feelings of tension and anxiety that occurs during teaching mathematical concepts, theories, and formulas or during problem solving” (p. 336). Hadley and Dorward (2011) found that anxiety about teaching math had a statistically significant negative correlation with student achievement. There is growing evidence about the potential negative influence of math anxiety on instructional practices, in particular, difficulty in explaining concepts due to struggles with working memory, spending less time on mathematics due to math avoidance, and using low cognitive demand tasks due to feelings of discomfort with concepts (Trice and Ogden, 1986; Karp, 1991; Brady and Bowd, 2005). In general, negative emotions (e.g., anxiety) tend to be accompanied by physiological and cognitive responses that interfere with mental and physical functioning (e.g., lack of focus and reduced working memory), and as such, teachers’ experiences of negative emotions related to teaching are considered non-conducive to effective teaching.

It is common in teacher emotion research for teachers’ emotional experiences to be defined around singular person–environment transactions within a teaching/classroom event which elicits a singular emotion, for example, anger or enjoyment. However, we consider teaching a highly complex activity centered around attainment of multiple, interconnected goals unfolding concurrently. Thus, teaching has the potential to provoke blended emotional experiences where multiple emotions are elicited.

### Sources of Teachers’ Emotions

One of the most emotional aspects of teaching is teachers’ relationships with their students, in particular, the emotional bonds and understandings they build with their students (Hargreaves, 1998; Day and Qing, 2009). Teachers also experience happiness, satisfaction, and pleasure in relation to students’ learning, especially when they are making progress and when they are responsive and cooperative during teaching (Emmer, 1994). Students’ interest and engagement in the content and observations of learning commonly elicited positive emotions across grade levels as advanced as university (e.g., Almeida and Mahoney, 2009). Teachers’ positive emotions are considered to have many positive personal and professional benefits; however, teachers’ enjoyment related to teaching has also been found to have student-related benefits (Frenzel et al., 2018). In particular, teachers who expressed higher levels of enjoyment related to teaching tended to align their teaching with a more student-focused approach to teaching (Stipek et al., 2001; Trigwell, 2012). Studies have also shown that teachers who have reasonable autonomy over their instructional time along with positive attitudes and emotions related to mathematics tended to spend more time in teaching mathematics (Lee, 2005; Russo et al., 2020). In contrast, teachers who tended to describe negative emotions related to teaching had more transmissive and teacher-centered instructional approaches that were thought to ensure content delivery (Trigwell, 2012) and tended to spend less time teaching math when possible (Trice and Ogden, 1987). Winograd (2005) suggests that high-quality teachers tend to describe feelings of enthusiasm, happiness, confidence, and satisfaction toward teaching.

Teachers’ experiences of negative emotions are often related to students’ behaviors (Hargreaves, 2000; Sutton, 2000). Chaves (2009) described teachers’ feelings of “impotence, sadness, frustration, nervousness, anger, irritation, and indignation” (p. 104) related to classroom disruptions initiated by students. In general, when teachers felt that they were not held in high regard by their students, this elicited negative emotions, such as anger, anxiety, and nervousness. Teachers also experience negative emotions, such as frustration, related to students’ thinking, generally when students are disengaged, inattentive, fall behind, or are not making progress (Reyna and Weiner, 2001; Russo et al., 2020). Both macro- and micro-contextual factors were also reasons of teachers experienced frustration and anger. Lack of support by colleagues, administration, and parents (Van Veen et al., 2005; Chaves, 2009), along with lack of time (Van Veen et al., 2005), or power dynamics results from age, class, or status (Candido-Ribiero, 2012). Anxiety and uncertainty tend to be experienced when teaching is considered to be difficult or complex and when teachers were not clear about the goals or expectations (Bullough et al., 1991).

### The Mathematical Context

There is reasonable consensus that high-quality mathematics teaching and learning occur in classrooms where teachers utilize cognitively challenging tasks that encourage problem solving and critical thinking and engage students in productive struggle (National Council of Teachers of Mathematics, 2014). Teachers often find constructing and managing classrooms that align with these features very challenging, and they often struggle due to a combination of factors, including mathematics-related beliefs (Cross Francis, 2015), low mathematical knowledge for teaching (Ball et al., 2008), and a range of contextual constraints such as time (Warshauer, 2015). High-quality mathematics teaching foregrounds student autonomy in creating and developing ideas, cognitive activation, and flexible and adaptive teaching that is responsive to students’ thinking, and the valuable role of struggle in meaningful learning (Stipek et al., 2001). Research would suggest that these particular instructional strategies tend to be associated with high levels of enjoyment (Stipek et al., 2001; Trigwell, 2012). In fact, Russo et al. (2020) found that teacher-reported enjoyment of mathematics was strongly positively related to teachers’ attitudes toward productive struggle in mathematics. Aligning the results with Pekrun’s (2006) control-value theory of emotions, they explained that teachers who value mathematics teaching and experience a sense of control during teaching will experience high levels of enjoyment and as such will encourage students’ productive struggles. On the other hand, teachers experiencing a low sense of control may feel anxious when teaching math, thus avoiding student struggle and resort to teacher-centered ways of instructing.

In the United States, the elementary mathematics classroom is often a “hot bed” of emotions for teachers and students. Elementary teachers often enter the profession because of their love of children and their inclination toward caring for them. Many tend to have negative associations with mathematics, including low efficacy related to mathematics, anxiety related to doing mathematics (Hembree, 1990; Hadley and Dorward, 2011), or anxiety related to teaching mathematics (Brown et al., 2011). These internal struggles are further exacerbated by pressures within the broader sociocultural context. Situated within the larger context of educational accountability, where students’ mathematics standardized test scores are used to determine both school and teaching quality, has increased stress for teachers (Hadley and Dorward, 2011). In many states, low test scores can have severe negative consequences for teacher pay, job security, and school sustainability and funding. It is within this context that we sought to broadly examine the relationship between teachers’ emotions and their mathematics teaching.

### Research Objective and Questions

Studies of teachers’ emotions are key because emotions have been found to influence motivation and subsequently behaviors (Mesquita et al., 1997), as well as attention, memory, thinking, and problem solving (Emmer, 1994). Emmer (1994) showed that teachers’ negative emotions, specifically anger and frustration, could distract teachers’ focus and attention from teaching. Additionally, high levels of anxiety can reduce working memory and is thought to be particularly detrimental for effective teaching (Trigwell, 2012). In contrast, positive emotions, such as joy and satisfaction, are suggested to provoke more teaching ideas and strategies (Sutton and Wheatley, 2003). These studies clearly establish a relationship between emotions, teachers’ cognitive and psychological processes, and behavior; however, what is less visible in the literature are descriptions of teachers’ emotional experiences in relation to teaching. Additionally, existing research presents emotional experiences as singular and discrete (e.g., anxiety) which do not seem to reflect the complex nature of teaching (Berrios, 2019).

In this regard, we focused on exploring teachers’ descriptions of their emotions both in anticipation of teaching (prior to teaching in pre-coaching conversations) and during teaching (through reflection during post-coaching conversations), to better understand teachers’ emotional experience through the complex activity of teaching. We also explored the reasons underlying their emotions and the relationship between these emotions and the quality of their mathematics instruction. We focus specifically on answering the following research questions:

(1) What emotions do elementary teachers describe in relation to their math instruction? How do they describe these emotions?
(2) What reasons underlie teachers’ emotions prior to and during reflection on their mathematics teaching?
(3) What is the relationship between teachers’ emotional experiences during teaching and the quality of their mathematics instruction?

## Methods

### Participants

The participants included seven teachers. All teachers taught elementary grades (grades kindergarten through sixth) and in schools that served high populations of students of color. Additionally, over 50 percent of students qualifying for free/reduced lunch, which is an indicator of low socioeconomic status. The teachers taught across three different schools within the same district in the Midwestern State in the United States. Table 1 includes additional information about the teachers.

**TABLE 1:** Demographic data on participants.

Teacher*	Position	Gender	Grade level	# of years of teaching
Bill	Special education teacher	Male	3rd	5
Sandra	Special education teacher	Female	Kindergarten	6
Laura	Elementary grades teacher	Female	4th	9
Anthony	6th-grade math teacher	Male	6th	15
Wilma	Elementary grade teacher	Female	2nd	10
Katie	Kindergarten teacher	Female	1st	19
Jessica	Elementary grade teacher	Female	2nd	5

*Names are pseudonyms.

### The Professional Development Program

These data were collected over the course of a year when teachers were involved in a professional development (PD) program designed to improve teachers’ mathematical knowledge for teaching and their instructional practices. The data is drawn from the 2nd year of the 2-year PD program. The format for the professional development was teacher coaching; specifically, all teachers were involved in five cycles (5) of coaching model referred to as Holistic Individualized Coaching. This model draws heavily on information about their mathematical knowledge for teaching, instructional quality, beliefs, professional identity, and emotions (see Cross Francis et al., 2019 for a full description). It involves six steps: (i) first, development of a general teacher profile, (ii) second, a pre-coaching discussion of a lesson to be coached, (iii) third, development of a content-specific mini teacher profile that considers all the constructs described above and information yielded from the pre-coaching discussion, (iv) fourth, pre-lesson support (if needed)–dependent on information in the mini profile, (v) fifth, in-class coaching where the coach is present to provide support during instruction, and (vi) sixth, the post-coaching conversation guided by the data from the videotaped lesson. Before the post-coaching conversation, the teacher and the coach watched the video-recorded lesson and identified three video clips they found interesting and useful for improving instruction. The post-coaching conversation was centered on discussing the instruction, planning, and thinking around the instruction visible in these video clips. Pre-coaching and post-coaching conversations were audio-recorded, and all instructions were video-recorded. For coaching to be effective, the coach–teacher relationship must be grounded in trust, respect, and transparency. The coach and the participants worked on developing a strong professional relationship during the 1st year of the program that allowed from honest and critical conversations around all aspects of the teaching experience during the coaching conversations. Participants were compensated with stipends and classroom resources for participation in the professional development program, but not for the study. Participants volunteered to participate in the study and were aware they could discontinue participation at any time without penalty as stated in the guidelines from the funding agency and the University’s Institutional Review Board.

### Data Sources

#### Audio Recordings From the Pre-coaching Conversation

A protocol was developed to guide the pre-coaching conversation, so they were consistent for each coaching cycle across teachers. The purpose of the conversation was multifold, including (i) to understand the teachers’ history with teaching the content of the to-be-coached lesson and their level of confidence related to teaching the topic, (ii) to understand teachers’ prior emotional experiences teaching the content and their current emotions related to the lesson and to students’ thinking in anticipation of teaching, (iii) to determine teachers’ knowledge related to the content and provide necessary support, and (iv) to plan the lesson collaboratively and discuss how the lesson will unfold. Questions in the protocol specifically served to elicit these data. For example, “how do you feel (e.g., anxious, frustrated, excitement) in anticipation of teaching the lesson?” All pre-coaching conversations were audio-recorded and transcribed. For this study, we specifically identified sections where teachers described their emotions and why they felt those emotions.

#### Audio-Recordings of Post-coaching Conversations

The post-coaching conversations also provided data about participants’ mathematical knowledge for teaching, emotions, efficacy, and their teacher role during the lesson. During these conversations, both the teacher and the coach discussed video clips each had selected from the coached lesson. The focus of these conversations was to identify instances of students’ thinking and discuss the teaching actions that served to support or hinder students’ thinking, as well as possible strategies to employ in future teaching to foreground mathematical thinking and reasoning. Regarding emotions, we specifically asked teachers to describe their state emotions related to four events: (i) teaching the lesson, (ii) students’ thinking and behavior, (iii) the video-recording, and (iv) the post-coaching conversations. For example, “how would you describe your emotions (e.g., pride, anxiety, shame, enjoyment, frustration) about teaching the lesson?” We also probed to determine the underlying reasons for the emotion. For this study, we specifically focused on identifying teachers’ statements about their emotions related to teaching the lesson and student thinking and behavior within the context of teaching, and why they felt the emotion(s). Both pre-coaching and post-coaching conversations were completed within a week of teaching the lesson.

For each of seven teachers, there were five coaching cycles for which there was one pre- and one post-coaching conversation (two conversations per cycle) where emotions about instruction were discussed. In total, there were 70 possible instances [70 = 7 (teachers) * 5 (cycles) * 2 (pre- and post-conversations)] where teachers described their emotions.

#### Video-Recordings From Coaching Session (MQI Scoring)

The videos served as the data source to determine the participants’ quality of instruction. We analyzed these videos using the MQI instrument to determine the quality of instruction along four core dimensions. The *MQI Instrument*^1^ (see Hill et al., 2008) was designed to provide a balanced, multidimensional perspective on mathematics instruction. The instrument provides a framework for examining mathematics instruction across four core dimensions, which include (i) common core-aligned student practices, (ii) working with students and mathematics, (iii) richness of the mathematics, and (iv) errors and imprecision. Within each of these four domains, there are several teaching characteristics to which points are applied differentially according to the level of instructional quality. These four dimensions are determined based on the selected segments of the videos that were the densest with mathematical activity and teacher–student interaction There is a fifth scale, *Whole Lesson Codes*, included in the MQI instrument that captures the knowledge and skills elaborated under the four core dimensions but applied to the entire lesson. For this analysis, we focused specifically on the scores *Whole Lesson Codes*. There are 10 items (e.g*., Teacher Attends to and Remediates Student Difficulty*) with a 5-point Likert scale.

### Data Analyses

#### Analysis of the Pre-coaching and Post-coaching Conversations

After the pre- and post-coaching conversations were transcribed, each teacher’s transcripts were assigned to each of the seven researchers. To answer the first research question, researchers focused on the sections of the transcripts where the teachers responded to the questions (i) how do you feel (e.g., anxious, frustrated, excitement) in anticipation of teaching the lesson? (pre-coaching conversations) and (ii) how would you describe your emotions (e.g., pride, anxiety, shame, enjoyment, frustration) about teaching the lesson, students’ thinking, watching your video, and discussing your teaching, and to what degree (low, medium, extreme)?

These sections were analyzed inductively as guided by the grounded theory approach (LeCompte et al., 1993; Corbin and Strauss, 2008). We reviewed the transcripts line by line, to identify words and phrases that reflected their emotions. Using Saldanþa’s (2016) descriptive coding method to identify the topic of data, these words and phrases were coded as “emotions.” During the second round of reading these segments identified with the “emotions” descriptive code, the words and phrases were coded using *in vivo* codes. *In vivo* coding entails taking out verbatim words or phrases from the data, so that we can stay close to the original meaning participants portrayed. For words or phrases that were not considered typical emotion words or in situations where the meaning was ambiguous, larger sections of the transcripts were coded to allow for interpretation of its meaning within context.

After all the transcripts had been coded in this way, two researchers reviewed all the transcripts for a second round of coding to enhance trustworthiness of data analysis. Where there were discrepancies, the two researchers discussed various sources of discrepancies such as definitional issues, context clarification, and various assumptions each researcher brings in. Through ongoing discussions to clarify those sources of discrepancies, agreement was reached. The lead researcher then compared all the codes across all the transcripts and sorted them into categories. There were initially three categories of positive emotions, negative emotions, and other emotions. A second researcher reviewed the categories for consistency and, along with the lead researcher, developed an additional category (blended emotions) and relevant subcategories. The development of these categories was then reviewed and discussed with the remaining five researchers until agreement was reached that those categories reflected our interpretations of the teachers’ descriptions of their emotions related to teaching and in anticipation of teaching. There was about 90 percent agreement. We then counted the frequency of emotions within each category.

To respond to research question two, researchers focused on the sections of the transcripts where participants described the reasons of the emotions they described. These descriptions primarily occurred during discussions of the video clips that were specifically selected by the teacher and in explaining why they felt the emotions they described. Similar to the inductive analytic approach we employed earlier, we first read the data segments multiple times to identify significant meaning units and then coded the units by labeling them with descriptive words or short phrases that preserved the essential meaning (Strauss and Corbin, 1998). Those codes across all participants’ data were then compared, contrasted, and sorted to generate categories and subcategories. This process of generating categories entailed searching for linkages in the emergent data structure by synthesizing connections and similarities among the codes. We then referred to the original transcripts to validate the categories. All seven researchers participated in these analytic steps and discussed questions or uncertainties that arose. We emphasized this researcher triangulation step to ensure that the results of the analyses were not biased due to individual researchers’ subjectivities, while generating insightful interpretations (Creswell and Poth, 2017). Once the agreed categories were generated, we counted the frequency of those categories and subcategories to represent and summarize the overall structure and dominance of patterns. We organized the outcome of the analysis in a table format, which was discussed in the findings section.

#### Videotaped, Coached Lessons (MQI Scoring)

The MQI instrument provides a framework for examining mathematics instruction across four core dimensions. Within each of these four domains, there are several teaching characteristics to which ratings are assigned differentially according to the level of instructional quality. Scores were assigned as not present–0; low–1; mid–2; and high–3. A fifth section, *Whole Lesson Codes*, assigns scores from 1 to 5; 1–low, and 5–high. With respect to scoring the five videos, first, two researchers watched each teacher’s instructional videos to score the instructional quality for the *Whole Lesson Codes* scale and assigned a score of one to five for the *Whole Lesson Codes* section of the instrument, following the guidelines provided in the MQI instrument. Second, both researchers selected 8 min of each video that focused on the segments of the videos that were the densest with mathematical activity and teacher–student interaction. Third, each researcher scored each of these 8-min video segments as High, Mid, Low, and Not Present for each aspect of the subcodes under the four dimensions. Numbers, as described above (not present–0, etc.), were assigned to the scores. Then, both researchers met, compared their scores, and reconciled any discrepancies. The average score from all the items in this dimension was determined. Finally, from the five videos, we selected the lesson from each teacher that had the highest average score (for the *Whole Lesson Codes* dimension) and examined the emotions the teachers described during that lesson. We selected the video with the highest score to see the nature of the emotions that teachers described for their best lesson. For the purposes of determining levels of quality, we considered an average score of > 4.0 on the *Whole Lesson Codes* section to be high-level instruction; scores between 3.0 and 4.0 mid-level; and scores below 3 low-level instruction.

## Findings

We organized the findings below by responding specifically to each research question.

### What Emotions Do Elementary Teachers Describe in Relation to Their Math Instruction?

To answer this research question, we analyzed data from the pre- and post-coaching conversations. Emotions described in the pre-coaching conversations should be interpreted as *emotions felt in anticipation of teaching.* Emotions described in the post-coaching conversations should be interpreted as *retrospective accounts of emotions felt during the teaching of the lesson*. Transcripts of these conversations were inductively analyzed, and results related to the teachers’ emotions described and the events that elicited the emotion are described in this section.

As described above, for the seven teachers, there were 70 possible emotional experiences–5 pre-coaching and 5 post-coaching conversations for each teacher. Of the 70 possible instances, teachers reported emotions on 60 instances. The 10 instances where no emotion was stated resulted for a range of reasons. One teacher, Wilma, asked not to have any pre-coaching conversations following the first coached lesson. Other teachers did not state an emotion although they were repeatedly asked about the emotion by the coach. In two instances, the teacher did not state an emotion or a physical feeling but an action. For example, when asked what emotion they felt while teaching the lesson, one teacher said “I feel like moving on.” This was not coded as an emotion. Table 2 (pre-coaching) and Appendix A (post-coaching) show the words teachers used to describe their emotions about math teaching.

**TABLE 2:** Teachers’ descriptions of their emotions during the pre-coaching conversations.

	Pre-coaching 1	Pre-coaching 2	Pre-coaching 3	Pre-coaching 4	Pre-coaching 5
Sandra	Not frustrated Confident (Mixed)	Okay Confident Overwhelmed (Mixed)	Good Positive (Blended-P)	Fine Comfortable (Neutral)	Anxious Good Okay Comfortable (Mixed)
Laura	Anxious Nervous (Blended–N)	Excited (Positive)	Excited (Positive)	Excited (Positive)	Excited (Positive)
Anthony	Excited (Positive)	Anxious Excited (Mixed)	Good Comfortable (Mixed)	Enjoyment Excited Concerned (Mixed)	Nervous (Negative)
Wilma	Concerned (Negative)	NONE	NONE	NONE	NONE
Katie	Indifferent Excited (Mixed)	Excited Curious Nervous (Mixed)	Anxious (Negative)	NONE	Comfortable (Neutral)
Jessica	Frustrated Anxious (Blended–N)	Not worried Comfortable Eager Surprised (Mixed)	NONE	Anxious (Negative)	Excited (Positive)
Bill	Excited Nervous (Mixed)	Excited (Positive)	Okay Not well prepared (Mixed)	Less Anxious (Negative)	Fine Hopeful (Mixed)

*P-positive; N-negative.*

### Discrete and Non-discrete Emotions

Some of the emotions teachers described were aligned with the discrete emotions described in the literature such as anxiety and frustration (Barrett et al., 2014; Frenzel, 2014). However, teachers described their emotions in several, non-typical ways. First, teachers described discrete emotions in the negative, to convey that they were not feeling that emotion. For example, teachers would state that they were “not nervous,” “not anxious,” or “not worried.” The valence of these emotions was not always clear so it was difficult to determine if “not anxious” was similar to calm or equal to enjoyment. Second, teachers described current emotional experiences in relation to emotions felt previously, for example, “less anxious (than last time)” which would be in reference to emotion they felt related to the last lesson. Third, teachers would state an emotion then position it to mean the opposite emotion, for example, stating they felt “anxious but in a positive way” which tended to mean that they were experiencing the physiological manifestations of the negative emotion [cf. butterflies on the stomach (Ganley et al., 2019)], but with positive affect. Another example stated by Bill in relation to teaching the fourth lesson was feeling “more comfortable in that uncomfortable feeling,” referencing a latent discomfort (sometimes described as anxiety) he tended to feel while teaching. In this instance, he described feeling less discomfort. Fourth, there were low occurrences of the typical discrete emotions: one instance of enjoyment (which were mixed); one instance of anger; and no instances of pride, shame, or guilt. However, we did note that there were several instances of excitement which teachers may align with enjoyment.

Fifth, teachers often describe their emotional experience using multiple emotions. In some instances, teachers expressed feeling one emotion related to teaching. This emotion was categorized as either positive (e.g., excited), negative (e.g., frustrated), or neutral. Neutral described feelings that were neither distinctively positive or negative, for example, calm or comfortable. Neutral emotions can be considered with low-valence, low-arousal in the circumplex model. We categorized emotions as blended emotions when the teachers described more than one emotion related to teaching a mathematics lesson. Table 3 (pre-coaching) and Appendix B (post-coaching) show these categorizations for the emotions described in the pre- and post-coaching conversations.

**TABLE 3:** Categorization of teachers’ emotions by type from the pre-coaching conversations.

	Positive	Negative	Blended (inclusive of Blended-Positive, Blended-Negative and Mixed)	Neutral
Sandra			• Not frustrated–Confident–Nervous • Okay–Confident–Overwhelmed • Good–Positive–Comfortable • Anxious–Good–Okay–Comfortable	• Fine–Comfortable
Laura	• Excited • (4 instances)		• Anxious–Nervous	
Anthony	• Excited	• Nervous	• Anxious–Excited • Good–Comfortable • Enjoyment–Excited–Concerned	
Wilma		• Concerned		
Katie		• Anxious	• Indifferent–Excited • Excited–Curious–Nervous	• Comfortable
Jessica	• Excited	• Anxious	• Frustrated–Anxious (in a positive way) • Not worried–Comfortable–Eager –Surprised	
Bill	• Excited	• Less anxious	• Excited–Nervous • Okay–Not well prepared • Fine–Hopeful	

### Blended Emotions

Blended emotions consisted of three combinations: (a) positive-blended emotions which described a combination of emotions that included all positive emotions, for example, when a teacher stated they felt enjoyment and pride related to teaching; (b) negative-blended emotions which described a combination of emotions that included all negative emotions, for example, when a teacher stated they felt stressed and frustrated related to teaching; and (c) mixed emotions described a combination of emotions that included positive, negative, and/or neutral emotions, for example, when a teacher stated that they felt anxious and excited about teaching. Mixed emotions were the emotions described most frequently–37 percent of emotions stated in the pre-coaching conversations and 79 percent of emotions stated in the post coaching conversations.

Teachers’ descriptions of blended emotions reflected the multiple and complex tasks that are involved in mathematics teaching and for which the teacher is continuously gauging the level of progress.

### How Blended Emotions Unfold

Teachers described how and why these emotions co-occurred in the coaching conversations. Some of these events were teacher-focused including the teachers’ comfort with the content of the lesson and their confidence in their ability to deploy strategies to engage students meaningfully with the concepts. Jessica’s emotional experience while teaching the fourth lesson demonstrated this well. Jessica described how she felt anxiety about the lesson, disappointment about her teaching, anxiety about student learning, and being comfortable with the lesson outcome (anxiety–disappointment–anxiety–comfortable–satisfied–proud).

“[Probably a little *anxious*] because I felt like at a point it was getting way too far off base, to where it’s like, OK, we have one error kind of on top of the other, and I don’t want this to confuse everybody…[it was *disappointing]* I didn’t give them enough information to discover on their own… I was a little bit *anxious* about teaching that because I didn’t know what questions they [students] would pose. And, you know, as much as you try to foresee the mistakes that they might make, so that you can ask a question to back that up, to help them think through it better. Those kind of things I was a little bit nervous about… Yeah, I felt *comfortable* with it, while I was teaching I felt like I asked some good questions too.”

Others were student-focused, related to the ebbs and flows of student engagement and learning as instruction unfolded. For example, related to the fifth lesson, Laura described complex mix of emotions (enjoyment–excited–concerned–nervous–relief–frustration–anger) that were mainly student-focused (also included some teaching(er)-focused emotions). She described,

“Well, I was very *nervous*for this lesson, and I don’t think it was necessarily because of the content and because of what it was that we were working on, but just simply because it was the second to last week of school, and they had already been kind of haywire for a solid week before that. And so I think that I was *nervous* for how it was going to go, and if we were really going to get anything out of it…I think maybe *relief*that they were actually doing what they were supposed to. *Relief* that they did seem to understand some of these things…I think that there was a little bit of *frustration* when I realized that, gosh, I’ve been plugging through this year trying to teach them about adding and subtracting and multiplying and dividing fractions, and some of them still don’t know what a fraction is. And so then that kind of turned a little bit toward *anger* at myself for not realizing this about my kiddos sooner.”

In several instances, the mixed emotions were related to both teacher-focused and student-focused events. With respect to the fourth lesson, Laura freaked out because she was not able to relate the measurement content that was the focus of the lesson to concepts she thought would appear on the standardized test. However, as the students engaged in the activity she was pleased because she saw that the students were able to grasp the concepts (freaked out–pleased–enjoyment).

“I *freaked out*…well, because we had been doing the trapezoids and the parallelograms and the triangles. I think that they’re more apt to see something about trapezoids and parallelograms and triangles on [standardized test] than they are a rectangle…and then while teaching, I was *pleased*because they did seem to enjoy it. I think it reassured me that it was decent because there were some things that they did need some clarification upon. And by going through that lesson, they were able to have that clarification. They were a little–they fairly quickly got what is area and what is perimeter, and it just reassured me that they did know this stuff.”

Mixed emotions were also related to the flow of the lesson. Sandra described her emotions related to teaching the fifth coaching lesson as “I guess I was a little *nervous* at first because I wanted them [students] to get it and understand what I was asking…I *felt okay*, because you [the coach] would chime in and you would get my thinking going and get their thinking going.” When the lesson began, she was nervous about teaching the content to her students because she was invested in her students understanding the content but was not sure she would be able to teach in ways to achieve this goal. However, her nervousness subsided as the coach provided input that stimulated both her thinking and the thinking of the students.

## What Reasons Underlie Teachers’ Emotions Prior to and on Reflection of Their Mathematics Teaching?

To respond to this research question, we inductively analyzed the transcripts of the pre- and post-coaching conversations of each teacher to determine the reasons underlying the elicited emotion. In what follows, we describe these reasons teachers described by emotion type. We also organized these data in Table 4.

**TABLE 4:** Reasons underlying teachers’ emotions.

Types of emotions	Time	Specific emotions	Reasons
Negative emotions	Pre-coaching	Anxiety	Uncertainty of students’ thinking and responses
		Nervousness	Fear that students might struggle to learn
	Post-coaching	Frustration	The need to differentiate content for a class with a wide range of competencies–the fact that one teacher has to teach for all students in the classroom
Positive emotions	Pre-coaching	Excitement	Some aspect of the concept they were teaching Concept being “new,” “fun,” “straightforward,” and “of personal interest”
	Post-coaching	Feeling good	Close alignment between the teachers’ anticipated student thinking and actual student thinking
Neutral emotions	Pre-coaching	Comfortable Fine	Knowing students’ capabilities related to the lesson
	Post-coaching	Comfortable	Knowing what students learned and didn’t learn during the lesson
Blended-positive emotions	Pre-coaching	Feeling good + Feeling positive	General sense of enjoyment of teaching
	Post-coaching	Feeling good + Not anxious	Students’ ability to complete classroom tasks
Blended-negative emotions	Pre-coaching	Anxious + Nervous	Teaching new concepts
		Frustrated + Anxious	Incorporating new curriculum to their teaching
	Post-coaching	Stressful + Frustrated	Not knowing how the lesson will unfold, and not seeing students’ learning quickly
Mixed emotions	Pre-coaching	Neutral (Okay, Comfortable, Fine, Not frustrated, Not worried) + Positive (good, positive)	Overall sense of competence, and confidence or enjoyment about teaching in general
		Neutral (Okay, Comfortable, Fine, Not frustrated, Not worried) + Negative (Anxious, Nervous, Overwhelmed)	Overall sense of competence, and various concerns such as teaching quality, students’ behavioral issues, students’ prior knowledge, and insufficient teaching preparation
	Post-coaching	Given the non-discernable pattern of mixed emotions for post-coaching conversation, various reasons associated with each emotion was explained in the text.	

### Negative Emotions

#### Pre-coaching

Anxiety is the dominated negative emotions teachers felt about teaching the coming lesson and expressed during the pre-coaching conversations. All the four teachers who experienced negative emotions about teaching the coming lesson expressed their anxious feeling. The major source for every teacher’s such negative emotions is related to the uncertainty of students’ thinking and responses. For examples, Anthony felt nervous to teach a lesson because he was afraid his students might mess up due to the complexity of the concept. Katie felt anxious because she was not sure if some of her students would be able to transition successfully from addition to subtraction. Jessica felt anxious because she could not predict what questions students might ask as she had never taught the concept using the approach before. Bill felt anxious about the possibility that his students would respond in ways he did not anticipate and he might struggle to address those situations and keep going.

#### Post-coaching

Frustration was the dominant negative emotion teachers stated feeling during teaching. Four of the five teachers who experienced negative emotions during the teaching of the lesson talked about their frustration. A major cause for their frustration is related to the need to differentiate content for a class with a wide range of competencies–the fact that one teacher has to teach for all students in the classroom. For example, Wilma talked about her frustration as there is the expectation that as a teacher you have to differentiate within your classroom and meet all of your students wherever they are and address their individual needs. Katie was frustrated by the fact that she had to do one-to-one interaction with 22 children to make sure all were reaching the learning goal. Jessica felt frustration because she did not understand why students were not understanding the content and why students not moving along as she expected. All these teachers experienced frustration related to teaching classes with cognitively diverse learners.

### Positive Emotions

#### Pre-coaching

All of the seven teachers who reported having positive emotions during the pre-coaching conversations expressed feeling excited Teachers’ feelings of excitement were primarily related to some aspect of the concept they were teaching. Five of the reasons stated were concept-focused and referred to the concept being “new,” “fun,” “straightforward,” and “of personal interest.” Bill was particularly excited because the concepts and the activities would be engaging for the students. Laura stated that she felt excited because she was interested in seeing how the students would react to the new approach of teaching the concept. She was using new activities and taking a discourse-centered approach to teaching the lesson. For another lesson, Laura described her excitement in relation to her teaching. She was a fifth-grade teacher and rarely used manipulatives. She was excited to include manipulatives in her lesson and was feeling very confident about her ability in supporting students throughout the lesson.

#### Post-coaching

There was one experience of positive emotion; Wilma stated she was “feeling good” during the post-coaching conversations and it was related to the close alignment between the teachers’ anticipated student thinking and actual student thinking.

### Neutral Emotions

Statements including the words “fine” and “comfortable” were used in three instances–two during the pre-coaching conversation and one during the post-coaching conversation. We interpreted fine and comfortable as similar emotions. Reasons for the elicitation of these emotions were all teaching-focused. Both Sandra and Katie stated they were comfortable based on their awareness of the student’s capabilities related to the concept of focus of the lesson. Katie stated “I’m comfortable. I think–I know where my kids are mathematically and I have an idea of how they will do based on the end of unit test.” Katie also stated that she felt comfortable during the post-conversation of another lesson. It was a student-focused emotion, and her feelings of comfort were related to both the students’ demonstrations of their mathematical ability (“I think they got the idea of computation”) and the struggles they experienced during the lesson (“I think they just struggled with the idea of subtraction. I think they knew that there was something to do with representing quantities and making less”).

### Blended Emotions

#### Pre-coaching

During pre-coaching conversations, teachers reported blended emotions in 15 instances. Out of 15, only 1 instance included blended-positive emotions that consisted of multiple positive emotions. For instance, Sandra reported her general sense of enjoyment of teaching with multiple positive emotions, “I *feel good*, I *feel positive* and I love the kids I work with, I really do. So, I feel okay, I expect things to go in the direction.” As an opposite case, two instances included blended-negative emotions that consisted of multiple negative emotions. Laura reported “anxious” and “nervous,” and Jennifer reported “frustrated” and “anxious.” Both instances were related to teaching new concepts or incorporating new curriculum to their teaching.

The remaining 12 instances included mixed emotions that consisted of various combinations of positive, negative, and neutral emotions. Teachers often expressed their overall sense of competence using neutral emotions such as “okay,” “comfortable,” and “fine” or the opposite of negative emotions such as “not frustrated” and “not worried.” Those neutral emotions were often used in conjunction with positive and/or negative emotions. Positive emotions in mixed emotions were most frequently related to teachers’ confidence or enjoyment about teaching in general. As pre-coaching conversation occurred prior to a classroom teaching, teachers’ overall disposition or affective responses about teaching seems to be reflected in their responses. Negative emotions in mixed emotions included “anxious,” “nervous,” and “overwhelmed” due to a range of concerns such as teaching quality (“I want to make sure they understand what I’m trying to get to”), students’ behavioral issues (“what is going on in the room”), students’ prior knowledge (“the ones that really don’t have the knowledge to know what I’m trying to explain”), and insufficient teaching preparation (“I have not my lesson plan ready yet.”). What is important to note is that teachers frequently reported a mix of these positive, negative, and/or neutral emotions during pre-coaching conversations, instead of reporting homogenously positive or negative emotions.

#### Post-coaching

Blended emotions were the most dominantly reported emotions during post-coaching conversations. Out of 35 possible instances, 25 instances included blended emotions. Similar to pre-coaching conversations, most frequently reported blended emotions were mixed emotions. Out of 25 instances of blended emotions, only one instance included blended-positive emotions and 1 instance included blended-negative emotions. For blended-positive emotions, Katie “felt good” and felt “not anxious” about what her students were able to do. In terms of blended-negative emotions, Bill reported “stressful” when he does not know how the lesson will go and also felt “frustrated” when he does not see improvement of students’ learning quickly.

Mixed emotions included various combinations of positive, negative, and/or neutral emotions. There were no distinctive patterns of combinations across participants or across coaching cycles, which might reflect situation-specific nature of teaching. Among mixed emotions, negative emotions related to teaching (*n* = 24) were most frequently reported. Out of 24, nine instances of negative emotions such as “frustrated,” “stressed,” “disappointed,” or “concerned” were reported due to challenges and struggles during classroom teaching, which did not meet teachers’ own expectation about teaching. For instance, Sandra felt “stressed” because “that class, is driving me crazy. So that’s hard. We just have all these things going on.” Similarly, Jennifer felt “disappointed,” because “I feel like the lesson I did before this I didn’t give them enough. I wanted them to discover, but I didn’t give them enough information to discover on their own.” Teachers also felt “nervous,” “anxious,” or “concerned” due to their general concerns about teaching quality and desires to improve teaching quality (*n* = 5). Jessica articulated her negative emotions related to her concerns of teaching quality, “I think the initial feeling of it coming. You’re freaking out, because–and then through this thing you’re like, OK, am I working OK? Am I asking good questions? Am I telling the student right, and you’re worried about behaviors, of course, coming out.” Teachers’ negative emotions (“anxious,” “scared,” “frustrated,” and “dislike”) were also related to uncertainty and adapting to newness due to change of curriculum or teaching a different grade level for the first time (*n* = 4). Laura who gets to teach 5th grade for the first time noted, “I know the beginning of this year, I was scared to come in and have to teach fifth grade math. I never taught fifth grade math, and I was under the impression that I wasn’t good at math myself.” In a few instances, teachers expressed negative emotions due to the tension and pressure to meet the standards (*n* = 3), and time management and pacing during teaching (*n* = 3).

Besides these reasons that elicited negative emotions as a part of mixed emotions, teachers also reported students’ lack of learning as major reasons for negative emotions such as disappointment, frustration, and anxiety (*n* = 12). Laura, for instance, felt frustrated, “when I realized that, gosh, I’ve been plugging through this year trying to teach them about adding and subtracting and multiplying and dividing fractions, and some of us still don’t know what a fraction is.” Student behavioral issues such as lack of attention and disruptive behaviors made teachers feel negative emotions (*n* = 5), along with pressure to prepare good lessons (*N* = 2) and lack of adequate teaching support (*n* = 1).

When teachers reported positive emotions as a part of mixed emotions, the most frequently reported reason was due to students’ learning and progresses (*n* = 22). Out of 22 instances, teachers felt positive emotions such as excitement, surprise, happy, and proud when they witnessed students’ learning and progresses (*n* = 13). Katie noted, “That made me pretty happy and pretty proud…because I did feel like they did well and that they were pretty strong in that.” Teachers also often reported positively “surprised” when students’ learning outcome was above their expectations. Anthony said, “What surprised me was the number of students who were able to get the answers correct. I was surprised…I had a lot of students who were able to answer those questions with those equation problems. So that surprised me.” It was not only the learning outcome that made teachers feel positive emotions, but also students’ learning processes (*n* = 9). Teachers felt “happy,” “pleased,” and “excited,” when students were engaged in the learning activities, enjoyed learning itself, worked together well, and actively participated in class. The next most frequently reported reason for positive emotions was related to classroom teaching (*n* = 17). Teachers reported a range of positive emotions such as “confident,” “excited,” “enjoy,” “felt good,” and “positive,” when they could help students’ thinking process, they could help students engaged in lesson, and new teaching methods went well, all of which contribute quality teaching. Bill’s comments show his positive yet complex emotions regarding his progress in teaching, “I felt more comfortable in that uncomfortable feeling. I don’t know how to explain it…I’m prepared. It’s just to know you’re still working toward something that’s good or better than what you do now.” Lastly, in two instances, teachers felt positive emotions when they are adequately prepared to teach.

Neutral emotions in mixed emotions were not as frequent as positive or negative emotions. However, teachers occasionally reported their general sense of competence in classroom teaching (e.g., “lesson went well”) using neutral terms such as “comfortable,” “calm,” and “relieved,” or denial of negative emotions such as “not nervous” and “not anxious” (*n* = 10). They also reported neutral emotions when students made progresses in learning (*n* = 4; “I felt comfortable with it [the learning outcome]”), confidence in content knowledge (*n* = 3; “I was not nervous about the content.”), and receiving support (*n* = 4; “I guess because I had already had that conversation with other people [team members], I was pretty comfortable with what I was bringing to them”).

Lastly, in terms of the mix of positive, negative, and neutral emotions, no distinctive patterns were found across participants or across coaching cycles. Instead of reporting singular discrete emotion during teaching, or a certain combination of emotions consistently, teachers reported a mix of multiple various emotions. This probably reflects the complexity of classroom teaching that comes with fluctuation of various emotions.

## What Is the Relationship Between Teachers’ Emotional Experiences During Teaching and the Quality of Their Mathematics Instruction?

Each teacher taught five lessons which were video recorded then scored using the MQI instrument. We examined the scores from the *Whole Lesson Codes* section of the MQI across the five lessons for each teacher. To compare teachers’ optimal level of instruction and the emotions felt, we selected the lesson that had the highest score (indicated in the first row of Table 5) and compared it with the emotions described in the post-coaching conversation for that lesson. These data are documented in Table 5.

**TABLE 5:** Comparison between teachers’ instructional quality and their emotions.

Teachers	Laura	Sandra	Wilma	Bill	Anthony	Jessica	Katie
Coaching Cycle	5	5	3	5	4	2	5
MQI	4.78	4.56	4.78	4.56	3.78	4.11	3.89
Emotions (Post)	Freaked out–Pleased–Enjoyment–Surprised	Nervous–Good–Surprised–Happy–Concerned	Frustrated–Sympathy–Positive	Not super nervous–Overall positive–More discouraged–Excited	Not nervous–Nervous–Surprised–Concerned	Disapproval of self–Proud–Worried	No anxiety–Comfort-Happy–Proud

All teachers experienced mixed emotions during their best mathematics instruction. We observed that all seven teachers described experiencing positive and negative emotions including teachers demonstrated high teaching quality (>4.0), as well as those at demonstrating lower levels of teaching (Anthony, Jessica, and Katie).

## Discussion

In this study, we examined teachers’ emotional experiences in anticipation of (pre-coaching conversations) and during reflection on teaching (post-coaching conversations) mathematics. We discussed the range of emotions teachers described in relation to teaching mathematics over five coaching cycles and any changes observed in both trait and state emotions, the underlying reasons for these emotions, and the relationship between teachers’ quality of instruction and their emotional experiences. The findings both aligned with and deviated from existing literature related to teachers’ emotions in four distinct ways. In what follows, we discuss these findings, implications for both work with teachers and future research on teachers’ emotions.

### Prevalence of Blended Emotions

First, unlike studies (e.g., Keller et al., 2014) that capture teachers’ emotions using approaches that foreground discrete emotions, we allowed teachers to provide self-reports of their own affective states. What this approach yielded were descriptions of a multiplicity of emotions. These emotions aligned to some extent with the list of most common discrete emotions described in the literature (e.g., anxiety, enjoyment), emotions included in the circumplex model of affect (e.g., frustration, calm), but also deviated in the ways teachers’ emotions have often been represented and described. In particular, teachers not only described singular positive (e.g., happy) and negative (e.g., disappointed) emotions; they also described neutral emotions (described as positive-valence, low-arousal) and blended emotions. We also observed that teachers’ experiences of blended emotions, the experience of multiple emotions related to a single event, were the most prevalent type of emotion both in the pre-coaching (in anticipation of teaching) and in the post-coaching (related to actually teaching the lesson) conversations. Within the three types of blended emotions, mixed emotions were most dominant–37 percent of emotions stated in the pre-coaching conversations and 79 percent of emotions stated in the post-coaching conversations. This finding has three meaningful implications.

One, it appears that teachers may not have prioritized reporting what is regarded as socially desirable emotions, which for teachers are positive emotions. Instead, given the prevalence of blended emotions, teachers seemed to have described their emotions in ways that were authentic to them. Additionally, they support the notion that teaching is indeed emotional work (Schutz and Zembylas, 2009) and that teaching is also complex work (Cross Francis et al., 2017, 2018; Berrios, 2019). Specifically, the presence of blended emotions seems to indicate multiple appraisals in preparation for teaching and as the lesson unfolded. This would authenticate that teachers’ work is indeed multifaceted and complex and illuminates the multiple aspects of classroom activity for which teachers set goals and track progress. Other researchers (e.g., Frijda et al., 1989; Watson and Clark, 1992) have also found that individuals rarely described feeling positive and negative emotions in isolation. They found it rare that individuals would describe the feeling of a specific positive or negative emotion without also feeling other positive or negative emotions (Posner et al., 2005). This aligns with categorizations of blended-positive, blended-negative, and mixed emotions, which shows that emotions are not experienced as isolated, discrete feelings; rather, they are ambiguous, overlapping experiences. In this regard, there is tremendous benefit in allowing individuals to label and describe their emotional experiences as it allows us to understand important aspects of their authentic emotional experiences that have not been previously captured in research (Barrett and Fossum, 2001). Third, emotions are elicited based on teachers’ appraisals about goal attainment. Therefore, given that teaching encompasses multiple goals, it follows then that teachers would experience multiple emotions. We observed that this co-occurrence of emotions could result from the appraisal of two goals related to teaching or multiple emotions could co-occur related to the same goal, seemingly simultaneously as in the case of Laura. We consider this a fruitful site for further research, specifically exploring how and when blended emotions occur and the role of time in these occurrences.

### Reasons Underlying Emotions

Second, unpacking the antecedents of the teachers’ emotions provided insight into the various elements of the classroom that teachers consider and attend to in anticipation of and during teaching. Reasons teachers stated for pre-coaching emotions seemed to be more generic than post-coaching emotions. Teachers stated reasons for post-coaching emotions were more specific and situational which seemed to be a function of talking about emotions related to hypothetical events prior to teaching, in contrast to post-coaching conversations where they were describing actual experiences. This has important implications for how professionals support teachers, in that it might be valuable for professional developers to assist and encourage teachers to be more explicit and robust in their hypothetical projections of future instructional events. In so doing, this may allow for the enactment of relevant and useful emotional regulation strategies when needed during teaching.

Third, we also observed that teachers’ most dominant negative emotions which were anxiety and frustration were in relation to students’ thinking and the task of finding effective ways to differentiate instruction for cognitively diverse learners, respectively. Excitement, which was the most frequently experienced positive emotion stated in both the pre- and post-coaching conversation was elicited mainly for teacher/teaching-centered reasons such as teaching the concept itself and using new resources to teach the concepts. Similar to positive emotions, neutral emotions had mainly teacher-focused reasons which were related to teachers’ feelings of competence in relation to teaching. In relation to blended emotions, negative emotions were most dominant as a component of the collective emotional experience and underlying reasons aligned with those of singular negative emotions, as well as with existing literature (Cross, 2009; Cross and Hong, 2012). These antecedents also included teacher/teaching-focused reasons similar to those for the positive and neutral components of blended emotions. There was no discernable pattern with respect to the type of emotion that would be elicited in response to a particular teacher–environment transaction. For example, teaching a concept in a new way could elicit a negative or positive emotion, for a range of reasons depending on the teacher, such as their feelings about the concept itself or students’ struggles with the concept. These findings support the notion that teachers’ emotions related to instructing mathematics lessons are strongly connected to teacher- and student-based aspects of the lesson.

### High-Quality Teaching Eliciting Mixed Emotions

Research suggests that high-quality teachers, those who align their teaching with student-focused strategies (Stipek et al., 2001; Trigwell, 2012), tend to describe feelings of enthusiasm, happiness, confidence, and satisfaction toward teaching (Winograd, 2005). In contrast, those who experience negative emotions related to teaching tend to enact more transmissive teaching strategies (Trigwell, 2012). The fourth insight was that we observed that teachers who demonstrated high-quality teaching experienced mixed emotions. When we examined the highest-quality teaching of each participant, emotions ranged from “freaked out” to “enjoyment”–all of these experiences were categorized as mixed emotions–the co-occurrence of positive and negative emotions. For the four teachers (Laura, Sandra, Wilma and Bill) who demonstrated the highest levels of teaching, having scores greater than 4.0 on the MQI, they all expressed mixed emotions. In fact, for all the teachers, when they taught their best lesson (whether it had a high MQI score or not), they expressed mixed emotions. The co-occurrence of negative and positive emotions suggests that the teacher was perhaps aware of and attending to multiple classroom goals during the class. This attentiveness to the goals may have led to the lesson being their best lesson, and this awareness during reflection may indicate that the teachers take a critical perspective to their practices, which will bode well for continued improvement.

Reasons for these emotions included concerns about their teaching and how the lesson would unfold, students’ thinking, students’ struggles to grasp the concepts and their level of engagement. We observed that in several cases although the antecedent to the emotion was similar, the emotion was different. For example, both Sandra and Bill had emotions related to students’ thinking–Bill experienced excitement and Sandra was shocked. This finding supports earlier research in that teachers who teach well do experience positive emotions related to teaching (Winograd, 2005), but they also experience negative emotions in relation to the same teaching event, i.e., mixed emotion. This appeared to be a function of how teachers appraised the events as they unfolded during the lesson and in relation to the lesson as a whole. Notably, in the post-coaching conversations, teachers stated negative emotions (e.g., nervous, concerned, anxious) but for important and positive reasons. For example, feeling anxious because there is the desire to teach well but feels there is a lot of uncertainty about how things will unfold.

Despite the fact that their teaching would be regarded as high quality based on the scores on the MQI instrument, during the lesson and on reflecting in the post-coaching conversations, the teachers did not always perceive that all aspects of the lesson went well. This perception of goal incongruence, that they did not or were not progressing toward their goal, related to certain aspects of the classroom activity (e.g., student learning), and goal congruent for other aspects (e.g., enactment of the task), seemed to have resulted in mixed emotions. This finding provides two key insights. First, the experience of negative emotions does not always negatively impact teaching quality. Second, instructional coaching has the potential to play an important role in helping teachers better calibrate their perception of their teaching with actual teaching quality.

## Theoretical, Methodological, and Practical Implications

The findings of this study have several theoretical, methodological, and practical implications that can serve to advance approaches to the study of teachers’ emotions. First, unlike other interview-based teacher emotion studies that focus on emotion incidents (Erb, 2002) or significant emotional episodes, as Sutton and Wheatley (2003) suggested, we examined teachers’ daily experiences of emotions in the classroom by considering the whole teaching session as a unit of analysis. More specifically, we investigated emotion in relation to content-specific classroom teaching focusing on teachers’ emotional experiences in relation to act of teaching mathematics. We think the use of video was particularly effective in teachers’ abilities to describe their emotional experiences as they were teaching mathematics. Second, focusing on teachers’ momentary emotional experiences (state emotions), rather than general feelings about mathematics teaching (trait emotions), provides insight into the complexity of the emotional experiences of elementary teachers in relation to mathematics. This provided a lens into the emotional fluctuations that teachers undergo during teaching that informs how teaching may unfold. As such, these data would be particularly useful for teacher support professionals (e.g., instructional coaches, teacher leaders, professional developers) to support teachers in developing strategies to effectively navigate the emotional terrain of classroom teaching. Third, we found it tremendously valuable to provide teachers with the space to inductively describe their emotional experiences using their own emotion-denoting words. Through the use of conversation stimulated by the use of video, we were able to capture the teachers’ lived emotional experiences that were not masked by the researchers’ orientations to emotion that can occur by solely using pre-developed surveys. Using this method allowed us to see the interconnectedness of emotions that is sometimes obscured by experimental and deductive methodologies.

## Conclusion

Despite the essential role emotions play in teachers’ lives, research on teachers’ emotions has not paid much attention on teachers’ state emotions in the context of daily teaching. In this study, we address this gap by focusing on elementary teachers’ emotions while preparing for teaching and during teaching, reasons that underlie these emotions, and the relationship between these emotions and the quality of their teaching. Findings showed that teachers reported a range of categories of emotions, several understudied in the field, namely, blended emotions, and often in non-typical ways (e.g., “not nervous,” “anxious but in a positive way”). One of these overlooked categories, blended emotions, was the most dominant category, which reflects the complex and multifaceted nature of active instruction. The inductive methodological approach of this study allowed participants to label and communicate their own emotional states, which showed the complex, overlapping, and ambiguous nature of emotions in the context of teaching. This is often masked by more deductive approaches to the study of emotions. This study contributes opening up the possibilities of diversifying teacher emotion research and shows the significance and usefulness of understanding teachers’ emotions.

## Data Availability Statement

The original contributions presented in the study are included in the article, further inquiries can be directed to the corresponding author.

## Ethics Statement

The studies involving human participants were reviewed and approved by Indiana University Institutional Review Board. The patients/participants provided their written informed consent to participate in this study.

## Author Contributions

DC designed the project and developed the conceptual framing of this manuscript along with JH. DC, JL, AE, and KL were involved in professional development and data collection. All authors analyzed data through triangulation and contributed to writing and editing this manuscript.

## Conflict of Interest

The authors declare that the research was conducted in the absence of any commercial or financial relationships that could be construed as a potential conflict of interest.

## Footnotes

1 https://cepr.harvard.edu/mqi

## Appendix A

**TABLE A1:** Teachers’ descriptions of their emotions during the post-coaching conversations.

	Post-coaching 1	Post-coaching 2	Post-coaching 3	Post-coaching 4	Post-coaching 5
Sandra	Nervous Worried Comfortable Scared Confident Frustrated (Mixed)	Concerned Comfortable Anxious Good (Mixed)	Confident Comfortable Frustrated (Mixed)	Stressed Excited Frustrated (Mixed)	Nervous Okay Surprised Happy Concerned (Mixed)
Laura	Disappointed Surprised Pleased and excited Excited and anxious (Mixed)	Surprised Scared Less nervous Excited Enjoyment Comfortable Pleased (Mixed)	Not scared Feeling good Comfortable Concerned Happy (Mixed)	Freaked out Pleased Enjoyment Surprised (Mixed)	Enjoyment Excited Concerned Nervous Relief Frustration (Mixed)
Anthony	Concerned Surprised (Mixed)	Good Less anxious Accomplished (Mixed)	Anxiety Relieved/calm (Mixed)	Not nervous Nervous Surprised Concerned (Mixed)	Good Excited Nervous Invested Surprised Concerned (Mixed)
Wilma	Frustrated (Negative)	NONE	Frustrated Sympathy Positive (Mixed)	Pretty good Good (Positive)	Excited Positive Disappointed (Mixed)
Katie	Comfortable (Neutral)	Concerned Surprised (Mixed)	Not anxious Good (Blended-P)	A little frustrated (Negative)	Comfortable Not anxious Happy Proud (Mixed)
Jessica	Frustrated Dislike Anxious Positive surprise (Mixed)	Disapproval of self Proud Concerned (Mixed)	Frustrated (Negative)	Anxious Disappointed Comfortable Satisfied Proud (Mixed)	Anxious Relief of anxiety Grateful Proud (Mixed)
Bill	Frustrated (Negative)	Stressed Frustrated (Blended-N)	Not felt comfortable (Negative)	Not terribly anxious Prepared More comfortable in that uncomfortable feeling Happy Relieved (Mixed)	Not super nervous Positive overall A little bit more discouraged Excited (Mixed)

## Appendix B

**TABLE A2:** Categorization of teachers’ emotions by type from the post-coaching conversations.

	Positive	Negative	Blended (inclusive of blended-positive, blended-negative, and mixed)	Neutral
Sandra			• Nervous–Worried–Comfortable–Scared–Confident–Frustrated • Concerned–Comfortable–Anxious–Good • Confident–Comfortable–Frustrated • Stressed–Excited–Frustrated • Nervous–Okay–Surprised–Happy–Concerned	
Laura			• Disappointed–Surprised–Pleased and excited–Excited and anxious • Surprised–Scared–Less • Nervous–Excited–Enjoyment–Comfortable–Pleased • Not scared–Feeling good–Comfortable–Concerned–Happy • Freaked out–Pleased–Enjoyment–Surprised • Enjoyment–Excited–Concerned–Nervous–Relief–Frustration	
Anthony			• Concerned–Surprised • Good–Less Anxious–Accomplished • Anxious–Relieved/Calm • Not nervous–Nervous–Surprised–Concerned • Good–Excited–Nervous–Invested–Surprised–Concerned	
Wilma	• Pretty good-Good	• Frustrated	• Frustrated–Sympathy–Positive • Excited–Positive–Disappointed	
Katie		• A little frustrated	• Concerned–Surprised • Not anxious–Good • Comfortable–Not Anxious–Happy–Proud	• Comfortable
Jessica		• Frustrated	• Frustrated–Dislike–Anxious–Positive surprise • Disapproval of self–Proud–Concerned • Anxious–Disappointed–Comfortable–Satisfied–Proud • Anxious–Relief of anxiety–Grateful–Proud	
Bill		• Frustrated • Not felt comfortable	• Stressed–Frustrated • Not terribly anxious–Prepared–More comfortable in that uncomfortable feeling–Happy–Relieved • Not super nervous–Positive overall–A little bit more discouraged–Excited	
				
